# War-related stress scale

**DOI:** 10.1186/s40359-024-01687-9

**Published:** 2024-04-15

**Authors:** Lenka Vargová, Bibiána Jozefiaková, Martin Lačný, Matúš Adamkovič

**Affiliations:** 1 Faculty of Education, University of Presov, Prešov, Slovakia; 2 Institute of Social Sciences, Centre of Social and Psychological Sciences, Slovak Academy of Sciences, Košice, Slovakia; 3 Olomouc University Social Health Institute, Palacky University, Olomouc, Czechia; 4 Institute of Political Science, Faculty of Arts, University of Presov, Prešov, Slovakia; 5 Faculty of Humanities and Social Sciences, University of Jyväskylä, Jyväskylä, Finland; 6 Faculty of Education, Charles University, Prague, Czechia

**Keywords:** War, War-related stress, Stressors, Mental health

## Abstract

**Background:**

The current war in Ukraine has affected the well-being of people worldwide. In order to understand how difficult the situation is, specific stressors associated with war need to be measured. In response, an inventory of war-related stressors including its short form, has been developed.

**Methods:**

A list of potential war-related stressors was created, and the content validity of each item assessed. The list, along with other validated scales, was administered to a representative sample of the Slovak population (effective *N* = 1851). Exploratory factor analysis, confirmatory factor analysis, convergent validity analysis and network analysis were carried out to determine the optimal scale (long and short form) focused on war-related stressors.

**Results:**

The full version of the scale consists of 21 items, further divided into three factors: society-related stressors, person-related stressors, and security-related stressors. The short version of the scale comprises nine items loaded onto one factor. These items cover concerns for one’s safety and future, access to necessities, potential worsening of the economic situation, and the risk of conflict escalation, including a nuclear threat. The results of the network analysis indicate that concern about escalation and fear of an economic crisis play a central role.

**Conclusions:**

The scale attempts to encompass a wide spectrum of areas that are affected by war and its potential consequences on individuals who reside outside the conflict zone. Given the complexity of the issue, researchers are invited to modify the scale, tailoring it to specific cultural, geographical, and temporal contexts.

## Background

The world had barely recovered from the COVID-19 pandemic when the Russian Federation invaded Ukraine in February 2022. According to the DSM-5, war is considered a significant stressor that meets the criteria of trauma. Trauma has been defined as “actual or threatened death, serious injury, or sexual violence” [1]. Exposure to war can thus be related to various mental health disorders such as anxiety, depressive disorders, trauma and stressor-related disorders (acute stress disorder or post-traumatic stress disorder), and addictive disorders [1]. According to WHO estimates, the prevalence of mental disorders in a conflict affected population stands at 22.1%; with a 13% prevalence of mild depression, anxiety, and PTSD, and a 4% prevalence of their moderate forms [2, see also 3].

Mental health problems and stress can also affect people living outside the conflict area [4, 5] and is caused by a number of war-related threats. Firstly, people’s sense of safety is disrupted with those living in surrounding countries and further afield fearing conflict escalation or nuclear war [6]. This is especially the case when the threat is definable and proximate [7]. The associated crisis can also have a global economic impact that involves higher inflation, supply chain disruptions, stock swings, reductions in investment and economic uncertainty [8]. People are thus affected by the worsening of the economic situation, potential lack of goods and commodities (even essential products), disrupted medical services, community consequences (e.g., functioning of public institutions, dealing with migration, etc.; [9] and the often difficult-to-predict development of the political situation [10]. The loss of purchasing power among citizens can mean subsequent risks to political stability as well [11]. According to the McKinsey Global Survey [12] on economic conditions, geopolitical conflicts remain the top-cited risk to global economic growth over the next 12 months, while inflation continues to be in second place.

Even populations that are not directly involved in conflicts face instability, fear and various worries which can contribute to a mental health burden [13]. Despite the scarcity of studies examining the mental health effects of war on people living outside conflict areas, the available evidence suggests the impairment of mental health (e.g., increased likelihood of depression and anxiety, PTSD, or a worsened quality of life) as a result of enduring stressors related to the war, consequential impacts and global threats [14–17]. The mental health impact of war has been observed in directly-affected Ukrainian citizens as well as in populations residing outside the conflict zone [18].

Civilians and refugees from war-affected areas facing complex traumas as well as those outside the conflict areas watching the war and suffering from helplessness and hopelessness are at risk for impaired mental health [19]. The variations of impairment may arise from different stress factors they have to face, emphasizing the importance of applying distinct interventions and policies [20]. Many countries can be faced with an increased need for mental health care as a result, and it is important to be aware of the situation and prepare the healthcare system for it [21, 22]. However, studies usually trace emotional reactions and the impact of war on people’s mental health retrospectively [23]. Furthermore, there is a dearth of measures that specifically focus on war-related stressors [6], and a scarcity of studies focused on this particular issue, especially in populations living outside the war zone [14]. The present study therefore aims to develop and validate a measure covering a wide spectrum of areas that are affected by war or an armed conflict and their (potential) consequences. These areas encompass the negative outcomes of the situation on people’s lives, which can be labeled as war-related stress.

## Methods

### Scale construction

The scale construction followed scale development guidelines [e.g., 24] that include (1) determining what the scale should measure and clarifying the construct’s content, (2) generation of an item pool and item quality check, (3) public and expert evaluation of the items’ content validity, (4) setting the measurement format, (5) a piloting and understandability check, (6) psychometric properties and (7) scale length reduction.

(1) Firstly, the areas of life which could be most affected by war were identified. The focus was on the areas that cause the most stress for people who are not living directly in a warzone or a military occupied country. The areas were identified and clarified using a combination of the following steps: (a) literature screening; (b) the research team assumed that the stress caused by war is similar to the stress related to the COVID-19 pandemic in many aspects. The primary inspiration was thus drawn from the validated measures of COVID-19 stress [e.g., 25, 26]; (c) a non-systematic screening of online discussion content (e.g., online journals, social networks) to potentially identify further stressful topics. (2) Based on this, an initial list of items was created, followed by a discussion to establish which areas were not covered sufficiently and which items were redundant or low on content validity. A similar discussion was held with two groups of undergraduate students. The list was continually updated throughout this process. The resulting version consisted of 22 statements. (3) The content validity of the 22 statements were independently rated by five experts (all experienced in quantitative research and survey designs; two of them also working in clinical practice). The ratings can be found at https://osf.io/f9kbx as well as in the analytic code. (4) Given the nature of the statements, a progressively increasing unidimensional response format was chosen with seven response options (ranging from 1 = No concerns/difficulties; 7 = Great concerns/difficulties). The utilized response format allows for easy gradation and discrimination between the options (increasing variance). Indeed, utilizing a wider scale (e.g., 1 to 100) would potentially increase measurement error and cognitive load on respondents. (5) Before the data collection, the items were piloted on a heterogeneous group of volunteers together with other scales administered in the survey^1^ (6 and 7). Besides validating the standard version of the scale, the aim was also to create and validate a short form of the scale (fewer than 10 items). A purely exploratory approach was utilized to examine the possible factor structure of the full scale. Given there was not only one solid a priori theoretical justification, a data-driven solution was preferred (none of the several predictions was found to be favorable) to see how the items would cluster into factors. A combination of a data-driven approach and evaluation of the content validity of the items were used to create a short version of the scale. Subsequently, both versions of the scale were cross-validated using a confirmatory approach. Furthermore, the convergent validity of the scales was examined by correlating them with a set of external criteria.

### Participants and data collection procedure

An online questionnaire was administered to a representative sample of Slovak inhabitants (*N* = 2127). The participants were sampled based on quota characteristics for gender, age, education, and region and were recruited by a Slovak agency specialized in online data collection. The data collection was part of a bigger project and thus included several scales. Ethical permission was granted by the Ethics Committee at the Faculty of Arts, University of Presov. Informed consent was obtained from each participant prior to the data collection. The study was conducted in compliance with the Declaration of Helsinki guidelines. The data was screened for missing values and careless respondents who were subsequently excluded (see https://osf.io/zut2m https://osf.io/zut2m?%20view_only=0a11ac371e3145c39c4b261e654ff001). The effective sample consisted of 1851 participants. The demographic characteristics of the sample are presented in Table 1.

**Table 1:** Demographic characteristics

Variable	Percentage or Mean ± SD
Gender (female)	53.81%
Age	44.36 ± 15.13
Partner status (married/in a relationship)	70.45%
Education (university degree)	27.66%
Residence (urban)	60.54%
Economic status (employed)	62.55%
Subjective socioeconomic status	5.39 ± 1.39
Equivalized household net income per month (in euros)	725 ± 445

*Note*: Subjective socioeconomic status was measured using a Cantril ladder; the scores ranged from 1 to 10 with a higher score indicating a higher subjective socioeconomic status. At the time of the data collection (March 2022), the average monthly gross income in Slovakia was 1212 euros (Statistical Office of the Slovak Republic, 2024), which works out at around 850 euros net

### Measures

The following measures were administered and used to examine the convergent/divergent validity. **Depression** was measured using the Quick Inventory of Depressive Symptomatology (QIDS-16-SR) [27]. The QIDS-16-SR consists of 16 items (each item represents a symptom of depression) covering nine depression symptoms from the DSM-5. For each item, the respondent expresses the degree of symptom severity over the last two weeks on a 4-point scale. Examples of areas covered include “Energy level“ or “General interest“. **Anxiety** was measured using the Generalized Anxiety Disorder Screener-7 (GAD-7; [28]) which consists of seven items. Participants are asked to rate how often they have been bothered by any of the presented problems over the last two weeks on a 4-point scale ranging from “not at all” to ”nearly every day”. Examples of these items include “Worrying too much about different things” or “Becoming easily annoyed or irritable”. The 10-item Perceived Stress Scale (PSS; [29]) was used to assess **subjectively perceived stress**. The PSS items are rated on a 5-point scale, ranging from ”never” to ”very often”. These include items such as “In the last month, how often have you felt nervous and stressed?”. **Sleep difficulties** were measured using the Insomnia Severity Index (ISI; [30]). The ISI consists of seven items which assess the different aspects of insomnia as well as assessing participants’ perception of nocturnal and diurnal symptoms of insomnia. These items include “How worried/distressed are you about your current sleep problem?”. **Loneliness** was measured using the short form of the Loneliness Scale (USL-8; [31]) which consists of eight items. Examples of items were “People are around me but not with me” or “I feel isolated from others”. The Brief Resilience Scale (BRS; [32]) was used to measure **resilience**. The BRS consists of six statements which are rated on a scale from 1 = ”strongly disagree” to 5 = ”strongly agree”. Examples of these items include “I tend to bounce back quickly after hard times”. **COVID-related anxiety** was measured by the COVID anxiety scale (CAS; [33]). The CAS consists of seven items that were answered on a 5-point scale, with a higher number representing higher levels of anxiety. This included items such as: “I have trouble relaxing when I think about COVID-19”. Items adapted from the COVIDiSTRESS survey [34] were used to measure **COVID-related stress**. The items in this questionnaire are formulated as statements assessing the presence of concerns and difficulties in various areas possibly affected by the COVID-19 pandemic, e.g., “difficulties in daily functioning” or “worrying about getting infected”. The statements were rated on a 7-point scale, with a higher number indicating a higher level of stress. For more detailed information about the measures see https://osf.io/bskr4 https://osf.io/bskr4?view_only=533d3324476e42018d661813b6ecace8. The descriptive statistics and reliabilities in the form of omega total coefficients are summarized in Table 2.

**Table 2:** Descriptive statistics and reliabilities of other scales

	M	SD	Potential range	ω_total_
Depression	1.59	0.51	1–4	0.90
Anxiety	1.68	0.66	1–4	0.95
Perceived stress	2.54	0.76	1–5	0.75
Insomnia	2.14	0.79	1–5	0.94
Loneliness	3.23	0.91	1–7	0.78
Resilience	3.16	0.61	1–5	0.85
COVID-related anxiety	2.14	1.02	1–5	0.93
COVID-related stress	3.38	1.23	1–7	0.94

*Note*: For all constructs, higher scores indicate higher levels of the construct

### Statistical analysis

The dataset was randomly split into three equal parts (each *N* = 617), with two of them being exploratory and one confirmatory.

1. Exploratory dataset 1 was used to determine the number of factors and find an optimal factor solution for the full version of the scale. The KMO index and a Bartlett’s test were computed to assess the factorability of the dataset. The KMO showed a very high sampling adequacy with an overall value of 0.97 (the lowest value = 0.94). The Bartlett’s test showed that the tested correlation matrix was not an identity matrix (χ^2^(231) = 9721.69, *p* <.001). A parallel analysis [35] was run to determine the number of factors. Based on the results, the optimal number of factors was found to be three. In the case of potential non-interpretability of the three-factor solution, several additional exploratory factor analyses were run with a hypothesized number of factors ranging from one to seven. The exploratory factor analyses were estimated using the weighted least squares method with a geominQ orthogonal rotation, and the variables treated as polychoric. In addition, a network analysis was used (e.g [36]) to examine how well the items were connected and which of them play a more central/peripheral role in the network. The network was estimated using the EBICglasso method and by setting the tuning parameter to 0.50 to get a sparse network. The items were screened for their factor loadings and cross-loadings, as well as for their strength parameter. An item was excluded if its highest factor loading did not exceed the threshold of 0.40 and had the lowest strength index at the same time. Given the exploratory nature of the study, items with high cross-loadings were not excluded but classified to the factor that included more similar items content-wise. The factor solution was then cross-validated on the confirmatory dataset.
2. Exploratory dataset 2 served to find an optimal short-scale version of the measure and for this, a one-factor solution was sought. In the first step of this process, five researchers (the authors and two senior psychology researchers) independently rated the content validity of each item. Given the high inter-rater agreement (ICC = 0.87, *p* <.001), the sum score of this evaluation was calculated for each item and the distribution of the scores was then examined. A cut-off of 40 was used (potential range of scores = 10–50) to select the items with the highest content validity. These items were then modeled using Confirmatory Factor Analysis (CFA), assuming a one-factor solution. The CFA was estimated using the weighted least square mean and variance adjusted method, treating the items as ordinal. The parameters of the model’s (mis)fit, the chi-square and approximate fit indices (CFI, TLI, RMSEA, SRMR), as well as the factor loadings, residual matrix, and modification indices were then examined. The potential sources of the model’s misfit were addressed to the level that was theoretically justifiable and the model was then re-estimated. The resulting solution was then cross-validated on the confirmatory dataset. In order to examine how these items are mutually connected, the items were further modeled using the network analysis with the exact same settings as previously described.
3. Confirmatory dataset 1 was used to cross-validate the findings. The three-factor model as well as the short-version one-factor model were estimated using CFA^2^. As before, the model-data fit was assessed based on the chi-square test and the approximate fit indices were examined. Two network models (corresponding to the list of items for the two factor models) were also computed to see how well the results replicated.

For all three datasets, the sum score of the measures was correlated with a set of external criteria^3^– the scores for depression, anxiety, general stress, insomnia, loneliness, resilience, COVID-related anxiety, and COVID-related stress.

Finally, two network analyses were computed to gain a better insight into how the specific indicators are mutually related. The first one included all the items involved in the resulting factor model from the first exploratory dataset, while the second one included the items of the short version of the scale as derived from the second exploratory dataset. Since network analysis is usually an exploratory technique, and has been reported as such, these networks were estimated on the whole dataset to achieve better stability and accuracy of the estimates.

The analyses were performed in R with *psych* [37], *lavaan* [38] and *bootnet* [39] serving as the main packages. The data and analytic workflow are documented at https://osf.io/jv2np/.

## Results

### Factor structure - full version

The parallel analysis suggested that the optimal number of factors is three. In comparison to other factor solutions, the three-factor model had sufficient psychometric properties (TLI = 0.95; RMSEA = 0.06, 95%CI [0.06, 0.07]; SRMR = 0.02) and was well-interpretable in terms of clarity of the factors. There was only one item (difficulty in distinguishing between true and fake information in the media) that had a factor loading below 0.40 as well as being low on centrality indices, especially strength. This item was thus excluded from the model and the three-factor model was re-estimated. The re-estimated factor model had about the same psychometric properties (both AFIs and factor loadings) as the previous version although no factor loading was smaller than 0.40. The items with high cross-loadings (> 0.40) were categorized to a factor that was more similar content-wise. In summary, the three factors are: (1) society-related stressors (items no. 7, 8, 9, 10, 16, 17, 18, 20), (2) person-related stressors excluding security stressors (items no. 3, 4, 5, 6, 11, 19, 21), (3) safety-related stressors (items no. 1, 2, 12, 13, 14, 15).

### Factor structure - short scale

After the raters’ evaluation of the content validity of the items, nine items (with a total score equal to 40 or more) were selected for the short form of the scale. Most of the selected items had had very high centrality indices when a network involving all the items was estimated in both the exploratory datasets. The selected items (item no. 1, 2, 4, 7, 12, 13, 14, 15, and 17) were then loaded onto a single factor. This model was estimated using CFA. With the exception of RMSEA, the model showed decent AFIs: CFI = 0.95, TLI = 0.93, RMSEA = 0.21 95% CI [0.19, 0.22], SRMR = 0.07, although it was still disconfirmed based on the chi-square test (χ^2^(27) = 737.20, *p* <.001). After examining the residual matrix and modification indices (MIs > 10), a covariance term was added between the following items: 1 and 2, 7 and 18, 13 and 14, 13 and 15, and 14 and 15. This improved the fit of the model (CFI = 0.99, TLI = 0.98, RMSEA = 0.11 95% CI [0.10, 0.12], and SRMR = 0.03) although the chi-square still indicated a significant model-data deviation (χ^2^(22) = 177.50, *p* <.001). There were no further modifications which could be theoretically well-justified. The estimated network of the items revealed that fear of the war spreading worldwide or to the country where the participant resided in addition to fear of an economic crisis, were the most central indicators of the war-related stress construct.

### Cross-validation of the results

The three-factor solution retained from the first exploratory dataset showed an unsatisfactory fit: χ^2^(186) = 2087.76, *p* <.001; CFI = 0.93, TLI = 0.92, RMSEA = 0.13 95% CI [0.12, 0.14], and SRMR = 0.06. The fit improved once the covariance terms (1 and 2, 7 and 18, 13 and 14, 13 and 15, and 14 and 15) were added: χ^2^(181) = 1582.91, *p* <.001; CFI = 0.95, TLI = 0.94, RMSEA = 0.11 95% CI [0.11, 0.12], and SRMR = 0.06 although it still did not reach satisfactory values (especially in the chi-square statistics). There were no further major, theoretically justifiable modifications which could have been done. As in the second exploratory dataset, the short form one-factor solution showed a very good fit in most of the AFIs (CFI = 0.99, TLI = 0.98, RMSEA = 0.11 95% CI [0.09, 0.13], and SRMR = 0.03) but still had a significant chi-square test (χ^2^(22) = 177.69, *p* <.001). All factor loadings and descriptive characteristics of the items are summarized in Table 3.

**Table 3:** Factor loadings and descriptive characteristics of the items

Item	Three-factor solution						Short form			Descriptives			
	Society		Person		Safety								
	E1	C	E1	C	E1	C	Raters’ score	E2	C	M	SD	Skew	Kurt
**1 Your own safety**	-	-	-	-	0.47	0.84	45	0.81	0.81	4.13	1.95	-0.12	-1.11
**2 Safety of your loved ones**	-	-	-	-	0.50	0.86	43	0.85	0.85	4.66	1.95	-0.45	-0.94
3 Work/school future	-	-	0.43	0.76	-	-	31	-	-	3.94	1.96	-0.05	-1.12
**4 Access to essential items (e.g., food, medicine, toiletries)**	-	-	0.43	0.85	-	-	43	0.75	0.78	4.14	1.91	-0.12	-1.07
5 Social relationships with your loved ones	-	-	0.74	0.76	-	-	24	-	-	3.39	1.93	0.31	-1.05
6 Social relationships in general	-	-	0.57	0.79	-	-	22	-	-	4.03	1.88	-0.09	-1.03
**7 Economic crisis in [country of residence]**	0.90	0.83	-	-	-	-	40	0.77	0.77	5.42	1.62	-0.94	0.14
8 Functioning of public institutions (e.g., governmental bodies, authorities, police)	0.48	0.77	-	-	-	-	29	-	-	3.94	1.85	-0.03	-1.00
9 Functioning of the healthcare system	0.69	0.83	-	-	-	-	28	-	-	4.66	1.84	-0.42	-0.85
10 Worsening of public health	0.49	0.83	-	-	-	-	24	-	-	4.38	1.83	-0.26	-0.92
11 Spending your free time	-	-	0.74	0.78	-	-	14	-	-	3.23	1.84	0.36	-0.89
**12 Uncertainty about the future development of the situation**	-	-	-	-	0.44	0.85	45	0.82	0.85	5.05	1.74	-0.71	-0.37
**13 Fear of the war moving to [country of residence]**	-	-	-	-	0.91	0.80	48	0.80	0.83	4.84	1.92	-0.53	-0.85
**14 Concern about the war moving to the EU or the world**	-	-	-	-	0.94	0.80	47	0.80	0.83	4.94	1.87	-0.60	-0.73
**15 Nuclear threat**	-	-	-	-	0.80	0.78	48	0.76	0.78	4.93	1.85	-0.59	-0.69
16 [Country of residence] coping with a migration wave	0.69	0.75	-	-	-	-	35	-	-	4.67	1.79	-0.39	-0.77
**17 Rising prices (inflation)**	0.89	0.67	-	-	-	-	42	0.68	0.61	5.95	1.37	-1.47	1.81
18 Lack of energy resources (e.g., gas, oil, electricity)	0.72	0.82	-	-	-	-	35	-	-	5.21	1.75	-0.84	-0.22
19 Worsening of your mental health (e.g., stress, anxiety, depression)	-	-	0.65	0.75	-	-	34	-	-	3.47	1.94	0.29	-1.07
20 Development of the political situation in [country of residence]	0.82	0.71	-	-	-	-	28	-	-	5.02	1.75	-0.63	-0.47
21 Quality of your life	-	-	0.50	0.85	-	-	37	-	-	4.19	1.86	-0.13	-1.02

*Note*: E1 = exploratory dataset 1; E2 = exploratory dataset 2; C = confirmatory dataset; Raters’ score potential range = 10-50; descriptive characteristics were obtained from the full sample

### Convergent validity

The correlations of the factors (three-factor model) and the total score (short-form model) from the respective exploratory and confirmatory datasets are available in Tables 4 and 5. The strongest positive relationship was detected between the war-related stressors (all three domains of the full version and the summary score of the shortened version) and (1) COVID-related stressors, (2) pandemic anxiety, (3) and general anxiety and depression. There were negative relationships observed in the case of resilience, which was also the smallest correlation (from − 0.11 to − 0.19 in the confirmatory dataset).

**Table 4:** Correlations of the three factors with external variables

	1	2	3	4	5	6	7	8	9	10	11
1 Society stressors	-	0.77	0.75	0.16	0.25	0.20	0.20	0.17	− 0.11	0.30	0.49
2 Person stressors	0.78	-	0.76	0.28	0.37	0.26	0.31	0.31	− 0.19	0.42	0.61
3 Safety stressors	0.75	0.76	-	0.19	0.30	0.23	0.20	0.18	− 0.17	0.34	0.48
4 Depression	0.18	0.31	0.26	-	0.71	0.59	0.62	0.42	− 0.38	0.40	0.38
5 Anxiety	0.26	0.40	0.35	0.69	-	0.69	0.60	0.42	− 0.36	0.51	0.48
6 Stress	0.19	0.24	0.23	0.57	0.63	-	0.48	0.34	− 0.21	0.40	0.44
7 Insomnia	0.24	0.35	0.30	0.60	0.56	0.44	-	0.37	− 0.27	0.44	0.44
8 Loneliness	0.11	0.25	0.14	0.44	0.44	0.40	0.40	-	− 0.26	0.35	0.43
9 Resilience	− 0.14	− 0.29	− 0.22	− 0.37	− 0.38	− 0.18	− 0.30	− 0.19	-	− 0.30	0.23
10 COVID-anxiety	0.34	0.48	0.45	0.48	0.53	0.41	0.48	0.32	− 0.32	-	0.66
11 COVID-stress	0.42	0.57	0.47	0.42	0.48	0.39	0.39	0.37	− 0.28	0.63	-

*Note*: Correlations from the exploratory dataset are below the diagonal, while correlations from the confirmatory dataset are above it

**Table 5:** Correlations of the single factor (short form) with external variables

	1	2	3	4	5	6	7	8	9
1 War stress	-	0.19	0.30	0.23	0.21	0.18	− 0.16	0.34	0.50
2 Depression	0.34	-	0.71	0.59	0.62	0.42	− 0.38	0.40	0.38
3 Anxiety	0.38	0.71	-	0.69	0.60	0.42	− 0.36	0.51	0.48
4 Stress	0.33	0.56	0.66	-	0.48	0.34	− 0.21	0.40	0.44
5 Insomnia	0.27	0.59	0.52	0.48	-	0.37	− 0.27	0.44	0.44
6 Loneliness	0.24	0.48	0.43	0.37	0.37	-	− 0.26	0.35	0.43
7 Resilience	− 0.24	− 0.42	− 0.44	− 0.26	− 0.25	− 0.28	-	− 0.30	− 0.23
8 COVID-anxiety	0.39	0.44	0.43	0.36	0.42	0.31	− 0.31	-	0.66
9 COVID-stress	0.46	0.45	0.46	0.39	0.47	0.39	− 0.32	0.68	-

*Note*: Correlations from the exploratory dataset are below the diagonal, while correlations from the confirmatory dataset are above it

### Network analysis

**Fig. 1:**
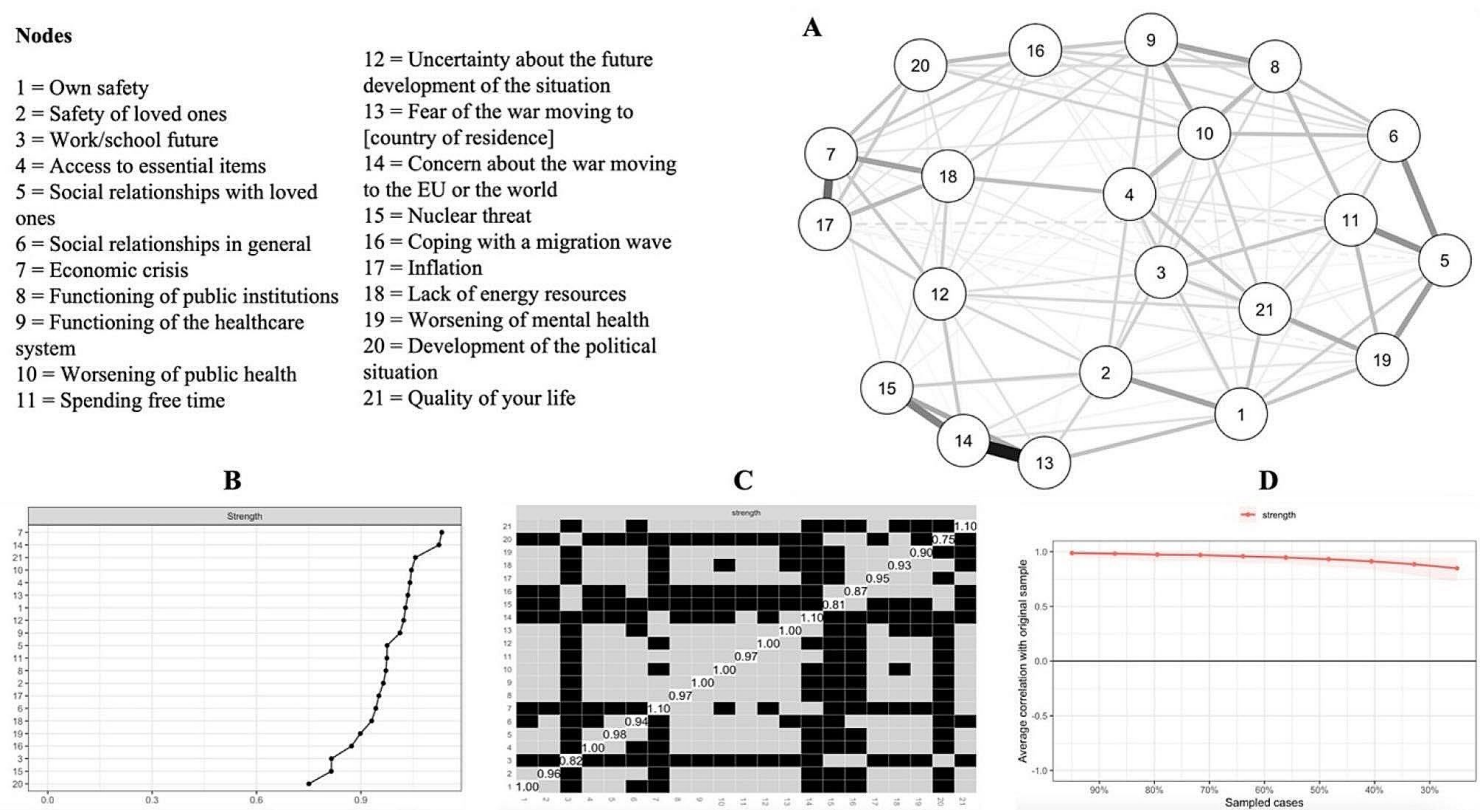
Visualization of the network of the full scale and items’ strength. A = Visualization of the network; B = Items’ strength (raw values); C = Differences in items’ strength; D = Stability of the strength indicator

**Fig. 2:**
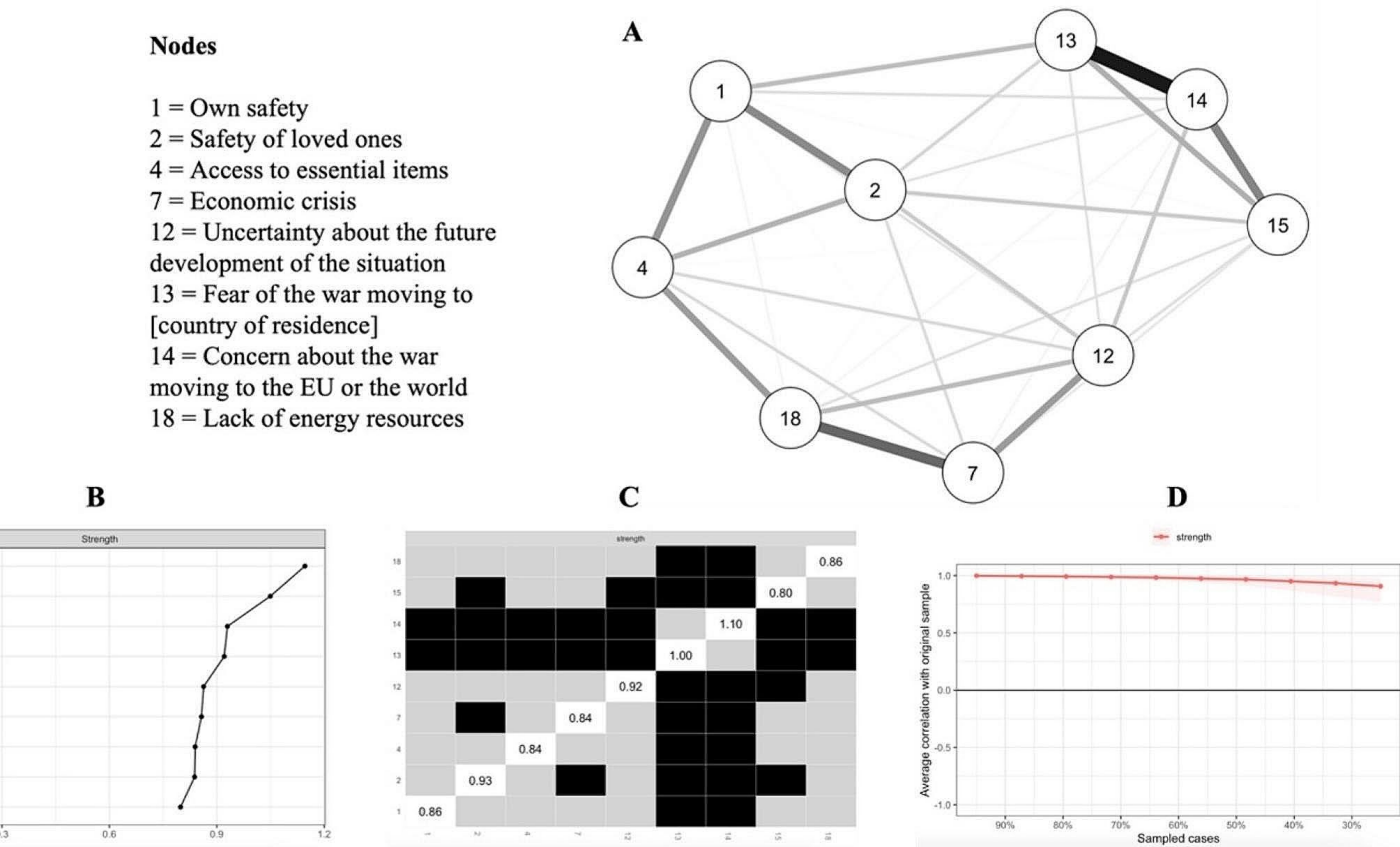
Visualization of the network of the short-form scale and items’ strength. A = Visualization of the network; B = Items’ strength (raw values); C = Differences in items’ strength; D = Stability of the strength indicator

Figures 1 and 2 show the visualizations of the networks, centrality indices, their stability, and the difference in the strength of the items. In the network that involved all 21 items, items no. 7 and 14 were the highest on the strength indicator, while items no. 20, 15, and 3 were significantly lower in strength compared to the rest of the items. In the network involving the short-scale items, items no. 14 and 13 had the highest strength indicator. Additional outputs of all the analyses can be found at https://osf.io/jv2np/.

## Discussion

After COVID-19, the war in Ukraine has been another crisis significantly affecting people’s mental health. This is especially the case if stressors accumulate (see [19, 20, 40, 41]). For a better understanding of the relationship between war and mental health, it is important to have adequate measures that can capture the different aspects of a military conflict as accurately as possible.

As such, an inventory of the most-pressing war-related stressors (at the time and considering the context) has been developed and validated. The full version of the inventory consists of 21 items that can be further divided into three factors: (1) society-related stressors (8 items); (2) person-related stressors (7 items) and (3) security-related stressors (6 items). The first factor, society-related stressors, consists of items related to the functioning of the country, such as management of the economic and migration crisis and the sufficiency of energy sources. The second factor, person-related stressors, covers items directly related to a person’s life (e.g., worries about the future, personal functioning, social relationships and quality of life). The third factor, security-related stressors, includes items related to safety issues, such as personal safety, potential escalation of the conflict, and nuclear threats.

The second goal was to develop a short version of the inventory that can be used to quickly assess perceptions of war-related stressors. This version consists of nine items loaded on to one factor. These items cover fear for one’s safety and future, access to basic needs, potential worsening of the economic situation, and escalation of the conflict, and the possibility of nuclear threat. The results of the network analysis indicate that concern about escalation, (i.e., the spread of the conflict to Europe/other countries) seems to be the central indicator for this construct. As with COVID-related stressors [26], fear of an economic crisis was also found to play an important role.

Overall, the inventory items in both the full and abbreviated versions align with previous findings and highlight the importance of addressing key stressors during wartime [6, 8, 9]. As in previous research [e.g., 42], the current study has demonstrated positive correlations between war-related stress and mental health issues (anxiety, depression), alongside negative associations with resilience. Despite high levels of perceived stress, Kurapov et al. [43] found surprisingly low scores of anxiety and depression among Ukrainians after 6 months of experiencing war. Notably, individuals who remained living in Ukraine achieved lower levels of anxiety and depression compared to those who had moved abroad, emphasizing the highly traumatic and stressful potential of both leaving one’s homeland and being displaced [44]. Similarly, studies working with Ukrainian refugees have also reported worsened mental health outcomes [e.g., 45]. A scale which is specifically focused on measuring war-related stress can assist in understanding the impact of military conflict on people’s mental well-being better. Similarly, it is important to identify specific stressors that are particularly important during times of war and can subsequently help in targeting specific and effective interventions.

### Limits and perspectives

As far as we know, this study is one of the first in creating a valid scale of war-related stress. However, there remain several caveats in it that should be addressed by further research. (1) Even though the items have been developed and selected with regard to several aspects (see the Scale construction section), it is not an ultimate list of items which can operationalize the war-related stress construct. As such, other researchers are invited to not only replicate the factor structure using these items but also to refine it, add new items, and examine potential factor structures using an updated version of the scale. (2) Another potential limitation is that the scale focuses on individuals residing outside the conflict area. For those living directly within the conflict zone, the stressors are likely to be of a different nature and more specific. In particular, civilians and refugees in war zones face immediate threats and losses due to direct exposure to traumatic events, whereas people living outside conflict areas are more concerned with potential threats [18]. Consequently, the origins of symptomatology, their manifestations, and the optimal interventions may vary between the two groups. One example of this could be direct exposure to traumatic events leading to PTSD. In contrast, stressors can be more context-dependent and subject to change over time for individuals living outside the conflict zone. They may diminish with the disappearance of potential stressors or with a decrease in their salience on social media [see 5, 46]. (3) The amount and intensity of stressors are likely to be more worrisome for people in nearby countries compared to those living further away [see 47]. The generalizability of the results should also be considered with caution due to geographical proximity. Proximity can amplify anticipatory worries about escalation, and fear can be heightened by the influx of refugees as these countries closely witness the impact of war on human lives [16]. (4) Cultural and community differences should also be considered in future (replication) studies. In recent years, the global population has faced a variety of crises such as wars, the COVID-19 pandemic and poverty, among others [48]. Factors such as preparedness, political, economic and societal conditions, as well as responses to these crises, can influence the nature and intensity of stressors across different countries. In Slovakia, the challenges posed by the pandemic followed closely by war, could have heightened people’s fears for themselves and their loved ones, as well as concerns about worsening economic conditions and quality of life. Therefore, further studies are needed to explore the generalizability of the findings beyond the context of Slovakia.

## Conclusion

Concerns about war, especially its potential escalation and the threats of nuclear conflict or economic downturn, can have long-term effects on overall health (e.g., [49]). This scale aims to cover a variety of factors affected by war and the possible repercussions on those residing outside conflict zones. Due to the complexity of the issue, researchers are invited to further refine and adapt the scale to suit different cultural, geographical, and temporal settings. The customization of the scale, especially with consideration for the proximity of populations to conflict areas, can offer essential insights and broaden our comprehension of the varied impacts of military conflicts. By enhancing our understanding of the effects of war-related stressors on population health, it is possible to not only contribute to reducing international and national tensions but also to supporting the pursuit of just and lasting resolutions to conflicts.

## Data Availability

The data and analytic code are freely available at https://osf.io/jv2np/.

## Footnotes

1 Note that the piloting process has not been sufficiently documented as it was not expected to publish this scale as a stand-alone paper at that time. The initial aim was to create a comprehensive list of relevant war-related stressors to map the experiences of the Slovak population, not to validate the measure or to group the items into factors. Please also note that the scale was part of a longer survey for the purposes of a longitudinal project focused on the mental health of Slovak inhabitants (APVV-20-0319).

2 With *N* = 617, alpha = 0.05, RMSEA_0_ = 0.04, RMSEA_A_ = 0.08, and DF = 186 (three-factor solution) / 27 (one-factor solution), the power to detect the difference between RMSEA_0_ and RMSEA_A_ converges to 100%.

3 With *N* = 617, alpha = 0.05, and using a two-sided test, the study design had an 80% power to detect a correlation coefficient of 0.11.
